# Influence of Implant-Specific Characteristics on Insertion Torque and Primary Stability: An Ex Vivo Comparison of Two Implant Systems with a Shared Macrodesign

**DOI:** 10.3390/dj14070446

**Published:** 2026-07-16

**Authors:** Peter Gehrke, Philipp Klose, Maria Julia Pietruska, Günter Dhom, Octavio Weinhold, Jörg Neugebauer, Paul Weigl, Robert Sader

**Affiliations:** 1 Department for Oral, Cranio-Maxillofacial and Facial Plastic Surgery, Johann Wolfgang Goethe University, University Hospital Frankfurt, 60596 Frankfurt, Germany; 2 Academic Teaching and Research Institution of the Johann Wolfgang Goethe University Frankfurt am Main, Private Practice for Oral Surgery and Implant Dentistry, 67059 Ludwigshafen, Germany; dr-klose@prof-dhom.de (P.K.);; 3 Private Practice, 88131 Lindau, Germany; maria.pietruska@gmail.com; 4 Department for Oral and Craniomaxillofacial and Plastic Surgery, University Hospital Cologne, University of Cologne Faculty of Medicine, 50931 Cologne, Germany; 5 Transfer Institute Management of Dental & Oral Medicine, Steinbeis University, 39104 Magdeburg, Germany; 6 Department of Postgraduate Education, Center for Dentistry and Oral Medicine (Carolinum), University Hospital, Goethe University Frankfurt, 60596 Frankfurt, Germany

**Keywords:** dental implants, implant surface, insertion torque, primary stability, resonance frequency analysis, ISQ, ex vivo study

## Abstract

**Objectives**: The aim of this study was to compare insertion torque (IT) and primary stability, assessed by resonance frequency analysis (RFA), between two implant systems with a shared macrodesign but different surface morphology and titanium grade in a standardized ex vivo porcine bone model. **Methods**: Sixty implants (OmniTaper EV and Xive; *n* = 30 per group) were inserted into standardized porcine rib bone blocks using identical osteotomy protocols. Peak insertion torque and primary stability, assessed by resonance frequency analysis (RFA), were recorded immediately after placement. Between-group differences were evaluated using appropriate statistical methods. **Results**: Bone density did not differ significantly between groups. OmniTaper EV implants demonstrated significantly higher insertion torque (28.2 ± 6.5 Ncm vs. 24.2 ± 6.2 Ncm; *p* = 0.020) and higher ISQ values (74.1 ± 6.0 vs. 69.8 ± 6.9; *p* = 0.013) than Xive implants. Mixed-effects analyses confirmed both findings after adjustment for clustering. No significant correlation was found between insertion torque and ISQ. **Conclusions**: Despite sharing a common implant design concept and standardized osteotomy preparation, the investigated implant systems demonstrated significant differences in insertion torque and primary stability. The findings indicate that implant-specific characteristics, including surface morphology and titanium grade, may influence implant–bone interface mechanics during insertion. However, their individual contributions cannot be distinguished within the present study design and should therefore be interpreted as the combined influence of implant-specific characteristics.

## 1. Introduction

Primary implant stability represents a fundamental biomechanical prerequisite for predictable osseointegration and long-term implant success and is particularly relevant when immediate or early loading protocols are considered. Insufficient mechanical stability at placement may result in micromovements at the bone–implant interface exceeding the critical threshold of approximately 100–150 µm, thereby impairing early bone healing and potentially leading to fibrous tissue formation instead of direct bone anchorage [1,2]. Consequently, substantial efforts have focused on identifying implant- and site-related factors that enhance mechanical engagement at the time of insertion.

Mechanical fixation immediately after implant placement is referred to as primary stability, whereas the stability achieved later through bone remodeling and regeneration is defined as secondary stability [3,4]. While macro- and microscale features primarily determine mechanical engagement and tissue-level healing, nanoscale modifications have been shown to enhance early cellular responses. These biological effects may promote early bone formation [2,3].

Bone density and implant macrodesign have consistently been identified as the dominant determinants of insertion torque and resonance frequency analysis (RFA)—derived implant stability quotient (ISQ) values [5,6,7]. However, when bone quality and implant geometry are controlled, surface-related characteristics may contribute to mechanical behavior at the implant–bone interface under otherwise standardized conditions. Surface roughness is commonly classified according to the Sa parameter into smooth (<0.5 µm), minimally rough (0.5–1 µm), moderately rough (1–2 µm), and rough (>2 µm) surfaces [8]. Surface characteristics are generated by manufacturing procedures such as grit blasting, acid etching, anodization, or coating techniques. These processes modify the physicochemical properties of titanium implants [9].

Modern implant systems typically use moderately rough surfaces generated by grit blasting and acid etching, as these topographies have demonstrated superior bone-to-implant contact and enhanced biological integration compared with turned surfaces [10]. According to the classification proposed by Wennerberg and Albrektsson [8], moderately rough surfaces exhibit an arithmetic mean roughness (Ra or Sa) between 1 and 2 µm. The Xive and OmniTaper EV implant systems (both Dentsply Sirona Implants, Hanau, Germany) were developed from the same implant design concept and share the same principal macrodesign and thread configuration. However, they differ in surface morphology and titanium grade. OmniTaper EV represents the evolutionary successor to Xive. The Friadent plus surface used on Xive implants is generated by grit blasting, acid etching, and neutralization (Ra 2.75 ± 0.46 µm), whereas the OsseoSpeed surface additionally undergoes a fluoride-related chemical modification (Ra ≈ 1.97 µm). Despite differences in manufacturing processes and physicochemical characteristics, both surfaces are classified as moderately rough [9,10,11].

Beyond their biological effects, implant surface characteristics may also influence the mechanical interaction between implant and bone during insertion. Although several studies have investigated associations between surface roughness and RFA-derived implant stability, current evidence suggests that surface roughness has only a limited influence on primary stability, whereas its biological benefits become more evident during osseointegration [12,13]. While the biological advantages of moderately rough surfaces are well established, their isolated influence on primary mechanical stability remains less clearly defined. From a tribological perspective, implant insertion represents a dynamic interaction between the implant surface and bone. Frictional forces and contact mechanics may influence insertion torque, whereas RFA primarily reflects the overall stiffness of the implant–bone complex rather than interfacial friction alone [14,15,16,17].

Previous comparisons of implant surfaces were frequently confounded by differences in implant geometry and surgical protocols [18,19]. Consequently, the independent contribution of implant-specific characteristics to primary implant stability remains insufficiently understood. The Xive and OmniTaper EV systems provide a suitable experimental model for such a comparison. They were developed from the same implant design concept and share the principal implant macrodesign and thread configuration. Both systems are placed using identical drilling instrumentation and manufacturer-recommended osteotomy protocols. They differ, however, in surface morphology and titanium grade. Therefore, the present ex vivo study aimed to compare peak insertion torque and primary stability, measured by resonance frequency analysis (RFA), between the two implant systems under standardized osteotomy conditions.

The null hypothesis was that no significant differences would be observed between the two implant systems with respect to insertion torque and ISQ values.

## 2. Materials and Methods

**Figure 1:**
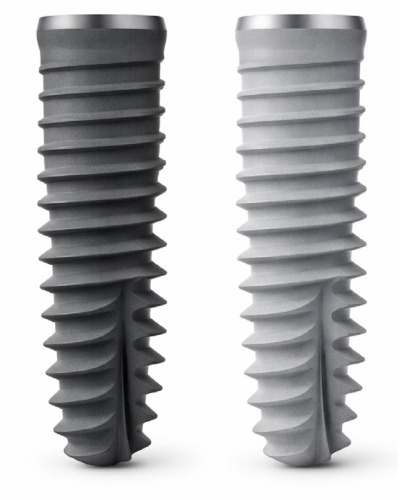
Comparison of the two implant systems evaluated. OmniTaper EV (**left**) and Xive (**right**) shown at identical scale and orientation.

In the present controlled ex vivo laboratory study, a total of 60 implants were placed in standardized porcine rib blocks (30 OmniTaper EV and 30 Xive implants). Fresh porcine thoracic rib segments were obtained as by-products of the food industry from skeletally mature domestic pigs processed for human consumption. No animals were euthanized specifically for this research. Only intact rib segments without visible cortical defects or anatomical abnormalities were included. Cortical bone thickness was not measured separately. All bone blocks were stabilized in a mechanical clamp during implant osteotomy preparation and subsequent implant insertion. Two implant systems from the same manufacturer (Dentsply Sirona Implants, Hanau, Germany) were compared. Thirty OmniTaper EV implants (OsseoSpeed surface) and 30 Xive implants (Friadent plus surface) were investigated (Figure 1).

No formal randomization of implant allocation was performed. Instead, both implant systems were distributed as evenly as possible across the available porcine rib specimens to minimize allocation bias. Group comparability was subsequently verified by quantitative assessment of local bone density. Some predefined implant positions within individual rib specimens were intentionally not used because of anatomical limitations (e.g., insufficient bone width or proximity to the rib end) that precluded standardized implant placement. This did not affect the predefined sample size or the balanced distribution of the 60 implants between the two implant systems and the investigated bone qualities (D3 and D4). All osteotomies and implant placements were performed according to the manufacturer’s recommended drilling protocols. The osteotomy preparation followed an identical sequential drilling protocol for both groups up to the final diameter of Ø 3.8 mm (Ø 2.0 → Ø 3.0 → Ø 3.4 → Ø 3.8), corresponding to the implant dimensions used in this study (Ø 3.8 × 11 mm). All drilling procedures were performed at a maximum speed of 1500 rpm under copious irrigation. Crestal preparation was additionally performed in both groups according to the manufacturer’s recommendations for low-density bone. The same drilling instruments and surgical drill set were used throughout the study, as both implant systems follow identical drilling protocols recommended by the manufacturer. According to the manufacturer’s technical specifications (Instructions for Use, IFU), Xive implants are manufactured from commercially pure titanium Grade 2, whereas OmniTaper EV implants are produced from commercially pure titanium Grade 4 and feature the OsseoSpeed surface modification.

All implants were inserted by a single operator using a surgical motor with real-time torque recording. Peak insertion torque (IT) was recorded in Ncm during implant placement, and primary stability was assessed immediately after insertion using resonance frequency analysis and expressed as implant stability quotient (ISQ). Bone quality was categorized according to the Misch classification (D3/D4) based on the macroscopic characteristics of the porcine rib specimens and was used for descriptive purposes only [20]. Local bone density was quantitatively assessed using CBCT-derived Hounsfield unit (HU) values obtained with a standardized region-of-interest (ROI) approach, as previously described for implant site assessment [21]. Accordingly, partial overlap between the measured HU values of D3 and D4 specimens may occur. Mean HU values were calculated within a 9-mm region of interest, and peak HU values of the cortical layer were recorded. Cortical bone thickness was not quantitatively measured in the present investigation.

Continuous variables are presented as mean ± SD. Normality was assessed using the Shapiro–Wilk test and Q–Q plots. Between-group comparisons were performed using Welch’s independent-samples *t*-test. To account for clustering of implant sites within porcine rib blocks, linear mixed-effects models were additionally performed as sensitivity analyses, including implant system as a fixed effect and bone block as a random intercept. Pearson correlations were calculated for bone density and outcome variables. Cohen’s d, mean differences, 95% confidence intervals, and *p*-values are reported. An a priori sample size estimation was based on effect sizes reported in previous experimental studies and feasibility considerations for controlled ex vivo designs (Cohen’s d = 0.65, α = 0.05, power = 80%) [22].

## 3. Results

### 3.1. Specimen Characteristics and Bone Density

A total of 60 implants were placed (30 OmniTaper EV and 30 Xive). No significant differences in bone density parameters were observed between the two implant systems. The mean trabecular density measured within the 9-mm region of interest was 471.7 ± 72.1 HU in the OmniTaper EV group and 480.0 ± 72.0 HU in the Xive group, while the cortical peak density was 2042 ± 82 HU and 2049 ± 80 HU, respectively (Table 1). Individual osteotomy-level values for Misch classification (D3/D4), mean HU, cortical peak HU, insertion torque, and ISQ are provided in Table 2.

### 3.2. Insertion Torque and Primary Stability (ISQ)

**Figure 2:**
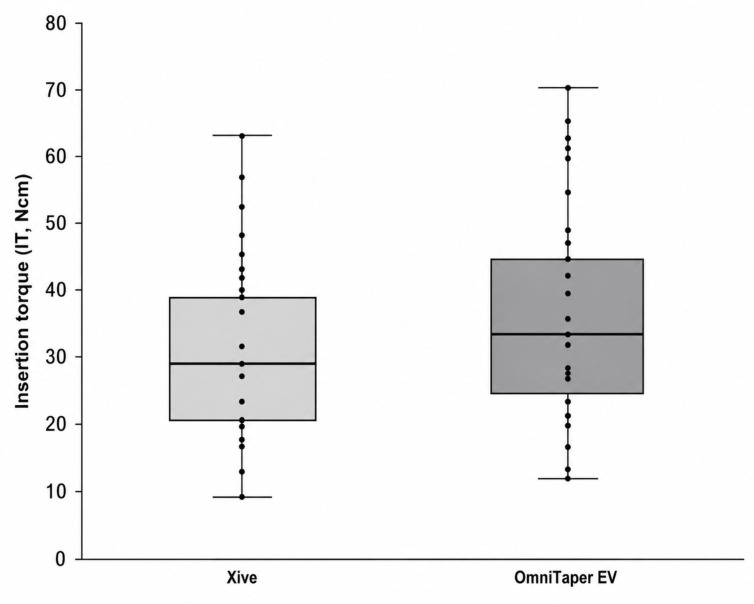
Box-and-whisker plot of insertion torque (IT) values for Xive and OmniTaper EV.

**Figure 3:**
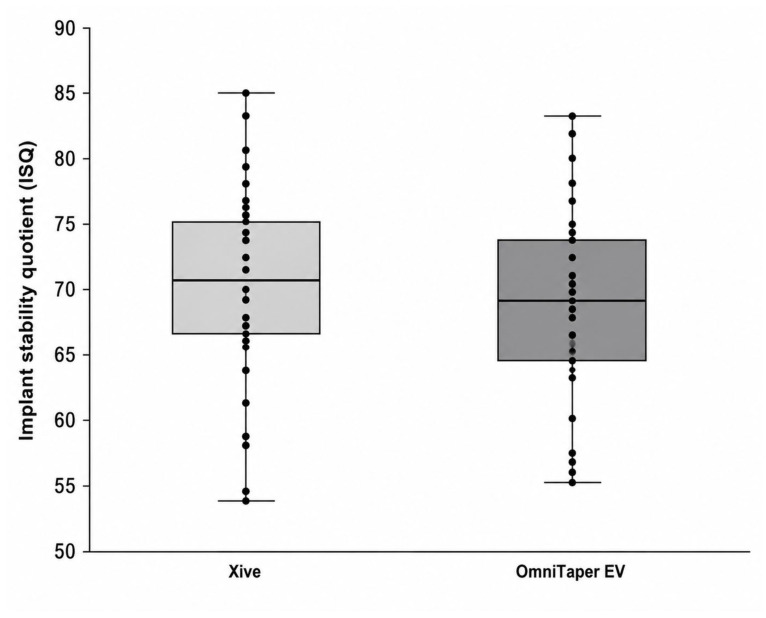
Box-and-whisker plot of implant stability quotient (ISQ) values for Xive and OmniTaper EV.

Peak insertion torque was significantly higher in the OmniTaper EV group than in the Xive group (28.2 ± 6.5 Ncm vs. 24.2 ± 6.2 Ncm; mean difference, 3.9 Ncm; 95% CI, 0.6–7.2; *p* = 0.020) (Table 3, Figure 2). Likewise, primary stability, measured by resonance frequency analysis (ISQ), was significantly higher in the OmniTaper EV group than in the Xive group (74.1 ± 6.0 vs. 69.8 ± 6.9; mean difference, 4.3 ISQ units; 95% CI, 0.9–7.6; *p* = 0.013) (Table 3, Figure 3).

To account for the clustering of implant sites within individual porcine rib blocks, linear mixed-effects models were additionally performed as sensitivity analyses. These analyses confirmed the findings obtained with Welch’s *t*-tests. The mixed-effects model confirmed a significant effect of implant system on both insertion torque (β = −4.29 Ncm for Xive relative to OmniTaper EV; 95% CI −6.95 to −1.64; *p* = 0.002) and implant stability (β = −4.13 ISQ units for Xive relative to OmniTaper EV; 95% CI −7.33 to −0.94; *p* = 0.011). Thus, accounting for the hierarchical structure of the data did not alter the statistical significance or direction of the observed between-group differences.

### 3.3. Relationship Between Insertion Torque and ISQ

**Figure 4:**
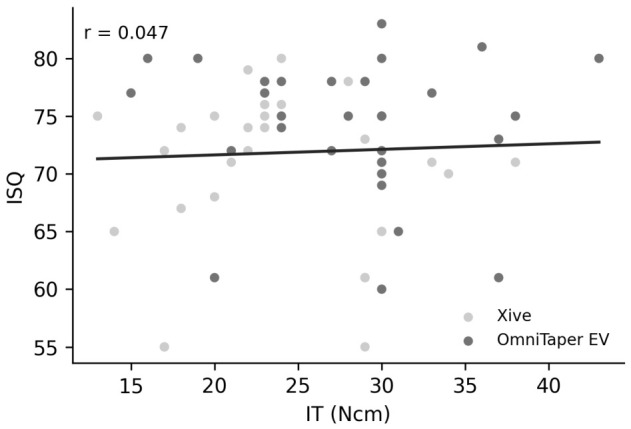
Scatter plot illustrating the relationship between peak insertion torque (IT) and implant stability quotient (ISQ). Each point represents one implant. The solid line indicates the linear regression fit. No significant correlation was observed (r = 0.047; *p* = 0.721).

Correlation analysis demonstrated no significant linear correlation between insertion torque and ISQ values (r = 0.047; *p* = 0.721) (Table 4; Figure 4). Similar non-significant correlations were observed when the implant systems were analyzed separately.

## 4. Discussion

In response to the growing adoption of immediate and early loading protocols, achieving sufficient primary implant stability has become a fundamental prerequisite in contemporary implant therapy. Implant stability develops through two distinct but interrelated phases: an initial mechanically driven phase immediately after implant placement, commonly referred to as primary stability, and a subsequent biologically mediated phase associated with bone remodeling and osseointegration, known as secondary stability [23].

The present study compared insertion torque and primary stability between two implant systems sharing the principal implant macrodesign and thread geometry under standardized osteotomy conditions. Both implant systems were placed using identical drilling instruments and manufacturer-recommended drilling protocols. Consequently, major confounding factors related to implant geometry and surgical preparation were minimized.

The principal implant-specific differences between the investigated systems were surface morphology, surface chemistry, and titanium grade (commercially pure titanium Grade 2 vs. Grade 4). Primary implant stability represents a multifactorial biomechanical phenomenon influenced by cortical bone morphology, bone density, surgical technique, implant macrodesign, and implant–bone contact mechanics. Therefore, the present findings should be interpreted within this multifactorial context. Accordingly, the present investigation was not designed to isolate the effect of implant surface modification alone. Rather, it compares two commercially available implant systems that share a common implant macrodesign while differing in several implant-specific characteristics, including surface morphology, surface chemistry, and titanium grade.

From a biomechanical and tribological perspective, primary stability may be understood as the result of contact mechanics occurring at the implant–bone interface during insertion. Frictional resistance, bone compression, and structural engagement between implant threads and surrounding trabecular and cortical bone contribute to the mechanical fixation achieved at placement. Within this context, bone density, cortical thickness, implant macrodesign, and surgical technique are generally regarded as the dominant determinants of primary implant stability [24,25].

Several experimental studies have demonstrated that bone morphology, particularly cortical thickness, strongly influences insertion torque values. Tabassum et al. reported that insertion torque increased with cortical thickness up to approximately 2 mm, whereas no further increase was observed in bone with thicker cortical layers [26]. Other investigations have likewise confirmed correlations between cortical bone thickness and implant stability parameters [27]. In addition to bone morphology, surgical technique has also been shown to influence primary stability. Undersized osteotomy preparation, particularly in low-density bone, may increase insertion torque and enhance mechanical fixation during implant placement [19,28]. To minimize the influence of such confounding variables, the present study followed the standard drilling protocol recommended by the manufacturer and used identical drilling instrumentation in both groups.

Beyond bone characteristics and surgical technique, implant macrodesign is another key determinant of primary stability. Progressive thread geometries and tapered implant designs have been associated with improved primary stability, particularly in compromised bone conditions [19,22,29]. While the influence of macrodesign has been extensively investigated, the specific contribution of implant surface microtopography to primary stability remains less clearly understood. From a biomaterial perspective, surface modification techniques primarily aim to enhance the biological processes occurring during the healing phase. Surface treatments such as grit blasting, acid etching, anodization, or chemical modification alter surface chemistry, roughness, and surface energy, thereby promoting osteoblast adhesion and bone formation at the implant interface [9,10]. Microscale roughness may influence implant–bone contact behavior at insertion, whereas nanoscale surface features affect protein adsorption and cellular signaling involved in early bone regeneration. Several studies have suggested that increased implant surface roughness improves osseointegration and implant success rates [13]. However, the relationship between surface roughness and primary mechanical stability remains controversial. A systematic review by Romero-Serrano et al. found that most available studies did not demonstrate a clear correlation between implant surface roughness and primary stability at the time of implant placement, although surface roughness appeared to contribute to secondary stability during healing [12]. Similar conclusions were reported by Javed et al., who noted that while rougher surfaces may improve long-term osseointegration, their direct influence on the initial mechanical stability of implants remains uncertain [15].

From a tribological perspective, implant insertion represents a dynamic contact interaction between titanium and bone under compressive and torsional loading [16,17]. Increased micro-roughness may increase the effective contact area and thereby influence interfacial contact mechanics between the implant surface and surrounding trabecular or cortical bone during implant insertion [14]. However, resonance frequency analysis (RFA) does not directly measure interfacial frictional forces but rather reflects the global stiffness of the implant–bone complex under lateral microvibration [15]. Thus, interfacial contact mechanics and structural stiffness represent related but distinct biomechanical phenomena. Within this context, the present ex vivo investigation demonstrated significantly higher insertion torque and ISQ values for OmniTaper EV implants under otherwise standardized conditions and comparable bone density. Although both implant systems were developed from the same implant design concept and shared the principal macrodesign, thread geometry, drilling instrumentation, and osteotomy protocols, the interpretation of these findings remains multifactorial. According to previous experimental investigations, cortical bone thickness may represent a major determinant of insertion torque and primary stability. Because cortical thickness was not quantitatively assessed in the present study, its influence cannot be excluded. In addition, the relatively small difference in insertion torque observed between groups may partly reflect experimental variation associated with biological specimens and osteotomy preparation tolerances.

Although the fluoride-modified OsseoSpeed surface has primarily been investigated with respect to its biological effects on early bone healing and osseointegration, its altered surface chemistry may also influence the mechanical interaction at the implant–bone interface during insertion. In addition to surface topography, physicochemical surface properties, including surface energy, wettability, and oxide layer composition, may affect frictional behavior and the transfer of insertion forces [16]. Consequently, differences in implant–bone interface mechanics observed immediately after implant placement may reflect not only the surface morphology but also chemical modifications of the implant surface. However, the specific contribution of fluoride-related surface modification to insertion mechanics remains insufficiently understood and warrants further experimental investigation. Likewise, differences in the mechanical properties of commercially pure titanium Grade 2 and Grade 4, including tensile strength and hardness, may also have influenced the biomechanical interaction between implant and bone during insertion. Therefore, the present findings should be interpreted as the result of multiple implant-specific factors acting simultaneously rather than as evidence of an isolated surface effect.

Previous studies have demonstrated favorable biological responses associated with chemically modified moderately rough implant surfaces during osseointegration [30]. However, whether such surface modifications directly influence primary mechanical stability at the time of insertion remains uncertain. Interestingly, OmniTaper EV implants also demonstrated significantly higher ISQ values despite the absence of biological healing in the present ex vivo model. Because resonance frequency analysis primarily reflects the stiffness of the implant–bone complex rather than osseointegration itself, these findings may indicate subtle differences in implant–bone interface behavior during insertion.

Furthermore, no significant correlation was observed between insertion torque and ISQ values. Although both parameters are widely used to characterize primary implant stability, they reflect different biomechanical properties. Insertion torque primarily represents the rotational resistance encountered during implant placement, whereas resonance frequency analysis assesses the stiffness of the implant–bone complex under lateral loading. Consequently, both parameters should be regarded as complementary rather than interchangeable measures of primary implant stability. The absence of a significant correlation in the present study is therefore consistent with the concept that mechanical fixation during implant insertion and structural stability after placement represent related but distinct biomechanical phenomena [4,5].

From a clinical perspective, increased primary stability may be advantageous for immediate or early loading protocols, which require sufficient mechanical fixation to prevent micromotion during the early healing phase. However, excessively high insertion torque may also increase compressive stress within cortical bone and potentially compromise local blood supply [14]. Overcompression of cortical bone has been associated with microdamage, impaired vascularization, and delayed bone remodeling in experimental models. Therefore, the potential benefits of increased primary stability should be balanced against possible biological risks, particularly in dense bone conditions. Consequently, the null hypothesis of no differences in insertion torque and ISQ values between the investigated implant systems was rejected.

Several limitations should be considered when interpreting the results of this study. First, the use of an ex vivo porcine bone model does not replicate the complex biological processes involved in osseointegration. Second, the absence of bone remodeling prevents conclusions regarding secondary stability and long-term implant success. Third, the mechanical properties of porcine rib bone may not fully reproduce the structural characteristics of human alveolar bone. In addition, cortical bone thickness was not quantitatively measured. Although local bone density was assessed using CBCT-derived Hounsfield units under standardized acquisition conditions, the accuracy of absolute HU values derived from CBCT remains limited because they may be influenced by scanner-specific acquisition and reconstruction parameters [31]. Nevertheless, all osteotomies were prepared using standardized drilling protocols and instrumentation, and variations in cortical morphology may still have influenced insertion torque and ISQ values. Furthermore, because the investigated implant systems differed not only in surface morphology but also in titanium grade (commercially pure titanium Grade 2 versus Grade 4), the independent contribution of each factor cannot be determined within the present study design. Accordingly, the observed differences should be interpreted as reflecting the combined influence of implant-specific characteristics, including surface morphology, surface chemistry, and titanium grade, rather than an isolated surface effect. Therefore, extrapolation of these findings to clinical situations should be made with appropriate caution.

Future investigations should further evaluate the relative contribution of implant surface characteristics, cortical bone morphology, and implant–bone interface mechanics to primary implant stability under varying surgical and bone conditions.

## 5. Conclusions

Within the limitations of this ex vivo porcine bone model, the investigated implant systems demonstrated differences in insertion torque and primary stability despite being developed from a common implant design concept and using standardized osteotomy preparation. Although differences in surface morphology, surface chemistry, and titanium grade may have contributed to the observed findings, the present study design does not permit discrimination of their individual contributions. Further experimental and clinical studies are warranted to elucidate the relative contributions of these implant-specific characteristics, together with cortical bone morphology and implant–bone interface mechanics, to primary implant stability and long-term clinical performance.

## Figures and Tables

**Table 1:** Specimen and bone density characteristics by implant system.

Implant System	Implants *n*	Mean HU (9 mm) Mean ± SD	Cortical Peak HU Mean ± SD
OmniTaper	30	471.7 ± 72.1	2042 ± 82
Xive	30	480.0 ± 72.0	2049 ± 80

HU = Hounsfield units. Mean HU refers to the average density measured over 9 mm as defined in the study protocol.

**Table 2:** Peak insertion torque (pIT), implant stability quotient (ISQ), values per drilling site, bone density, and Hounsfield Unit (HU).

Osteotomy ID	Block ID	Drill Site	Bone Type	Mean HU	Cortical Peak HU	Implant System	pIT (Ncm)	RFA (ISQ)
B1-S1	1	1	D3	470	2050	Xive	30	70
B1-S2	1	2	D3	470	2050	Xive	30	71
B1-S3	1	3	D3	470	2050	Xive	30	65
B1-S4	1	4	D3	470	2050	Xive	30	65
B1-S5	1	5	D3	470	2050	OmniTaper	30	80
B1-S6	1	6	D3	470	2050	OmniTaper	30	60
B1-S7	1	7	D3	470	2050	OmniTaper	30	70
B1-S8	1	8	D3	470	2050	OmniTaper	30	69
B2-S1	2	1	D3	480	2100	OmniTaper	30	75
B2-S2	2	2	D3	480	2100	Xive	23	75
B2-S3	2	3	D3	480	2100	OmniTaper	29	78
B2-S4	2	4	D3	480	2100	Xive	29	73
B2-S5	2	5	D3	480	2100	OmniTaper	43	80
B2-S6	2	6	D3	480	2100	Xive	28	78
B2-S7	2	7	D3	480	2100	OmniTaper	24	78
B2-S8	2	8	D3	480	2100	Xive	33	71
B2-S9	2	9	D3	480	2100	OmniTaper	27	72
B3-S1	3	1	D4	430	1950	Xive	17	55
B3-S2	3	2	D4	430	1950	OmniTaper	24	75
B3-S3	3	3	D4	430	1950	Xive	22	72
B3-S4	3	4	D4	430	1950	OmniTaper	16	80
B3-S5	3	5	D4	430	1950	Xive	20	75
B3-S6	3	6	D4	430	1950	OmniTaper	30	75
B3-S7	3	7	D4	430	1950	Xive	29	55
B3-S8	3	8	D4	430	1950	OmniTaper	37	73
B4-S1	4	1	D3	600	2150	Xive	18	67
B4-S2	4	2	D3	600	2150	OmniTaper	30	71
B4-S3	4	3	D3	600	2150	Xive	20	68
B4-S4	4	4	D3	600	2150	OmniTaper	30	72
B4-S5	4	5	D3	600	2150	Xive	24	76
B4-S6	4	6	D3	600	2150	OmniTaper	27	78
B4-S7	4	7	D3	600	2150	Xive	22	79
B4-S8	4	8	D3	600	2150	OmniTaper	24	74
B5-S1	5	1	D3	500	2080	Xive	17	55
B5-S2	5	2	D3	500	2080	OmniTaper	21	72
B5-S3	5	3	D3	500	2080	Xive	14	65
B5-S4	5	4	D3	500	2080	OmniTaper	20	61
B5-S5	5	5	D3	500	2080	Xive	13	75
B5-S6	5	6	D3	500	2080	OmniTaper	23	78
B5-S7	5	7	D3	500	2080	Xive	23	76
B5-S8	5	8	D3	500	2080	OmniTaper	28	75
B6-S1	6	1	D4	370	1920	Xive	18	74
B6-S2	6	2	D4	370	1920	OmniTaper	23	77
B6-S3	6	3	D4	370	1920	Xive	17	72
B6-S4	6	4	D4	370	1920	OmniTaper	19	80
B6-S5	6	5	D4	370	1920	Xive	21	71
B6-S6	6	6	D4	370	1920	OmniTaper	33	77
B6-S7	6	7	D4	370	1920	OmniTaper	30	83
B6-S8	6	8	D4	370	1920	Xive	24	80
B6-S9	6	9	D4	370	1920	OmniTaper	37	61
B7-S1	7	1	D3	550	2120	Xive	29	61
B7-S2	7	2	D3	550	2120	OmniTaper	31	65
B7-S3	7	3	D3	550	2120	Xive	29	61
B7-S4	7	4	D3	550	2120	OmniTaper	38	75
B7-S5	7	5	D3	550	2120	Xive	38	71
B7-S7	7	7	D3	550	2120	Xive	34	70
B8-S3	8	3	D3	400	2000	OmniTaper	15	77
B8-S4	8	4	D3	400	2000	Xive	23	74
B8-S5	8	5	D3	400	2000	Xive	22	74
B8-S6	8	6	D3	400	2000	OmniTaper	36	81
Total Xive (*n* = 30); OmniTaper EV (*n* = 30)								

pIT = peak insertion torque; ISQ = implant stability quotient; HU = Hounsfield units. Misch classification reported as D3 or D4.

**Table 3:** Insertion torque and primary stability (ISQ): descriptive statistics, effect sizes, and results of between-group comparisons.

Outcome	Xive (Mean ± SD; Range)	OmniTaper EV (Mean ± SD; Range)	Mean Difference (95% CI)	Cohen’s *d*	Welch’s t-Test (*p*)	Mixed- Effects Estimate β (95% CI)	Mixed- Effects Model (*p*)
Peak insertion torque (Ncm)	24.2 ± 6.2 (13.0–38.0) *n* = 30	28.2 ± 6.5 (15.0–43.0), *n* = 30	3.9 (0.6 to 7.2)	0.62	0.020	−4.29 (−6.95 to −1.64)	0.002
Primary stability (ISQ)	69.8 ± 6.9 (55.0–80.0) *n* = 30	74.1 ± 6.0 (60.0–83.0), *n* = 30	4.3 (0.9 to 7.6)	0.66	0.013	−4.13 (−7.33 to −0.94)	0.011

Values are presented as mean ± standard deviation (SD) with ranges in parentheses. Mean differences are presented as OmniTaper EV minus Xive. Welch’s independent-samples *t*-tests were used for the primary analysis. Linear mixed-effects models included implant system as a fixed effect and bone block as a random intercept and were performed as sensitivity analyses. Cohen’s d values represent standardized effect sizes. CI = confidence interval; ISQ = implant stability quotient.

**Table 4:** Pearson correlation between IT and ISQ.

Group	N	Pearson r (pIT vs. ISQ)	*p* Value
**Overall**	60	0.047	0.721
**Xive**	30	−0.018	0.925
**OmniTaper EV**	30	−0.092	0.629

Pearson correlation for pIT vs. ISQ. *p* values based on two-sided tests.

## Data Availability

The data presented in this study can be made available on reasonable request to the corresponding author.

## Abbreviations

The following abbreviations are used in this manuscript:

ISQ	Implant stability quotient
RFA	Resonance frequency analysis
pIT	Peak insertion torque
HU	Hounsfield Unit
ROI	Region-of-interest
SD	Standard deviation
